# Supplementary material for: The CENP-A centromere targeting domain facilitates H4K20 monomethylation in the nucleosome by structural polymorphism
Source: Nat Commun. 2019 Feb 4;10:576. doi: 10.1038/s41467-019-08314-x (PMC6362020; doi:10.1038/s41467-019-08314-x)
Supplement: Supplementary file 1 — Supplementary Information [file 41467_2019_8314_MOESM1_ESM.pdf]

Supplementary Information

The CENP-A centromere targeting domain facilitates H4K20 monomethylation in the nucleosome by structural polymorphism

Yasuhiro Arimura[1,2,6], Hiroaki Tachiwana[2,3,6], Hiroki Takagi[1,2,6], Tetsuya Hori[4], Hiroshi Kimura[5], Tatsuo Fukagawa[4], and Hitoshi Kurumizaka[1,2]

[1]Laboratory of Chromatin Structure and Function, Institute for Quantitative Biosciences, The University of Tokyo, 1-1-1 Yayoi, Bunkyo-ku, Tokyo 113-0032, Japan. [2]Graduate School of Advanced Science and Engineering, Waseda University, 2-2 Wakamatsu-cho, Shinjuku-ku, Tokyo 162-8480, Japan. [3]The Cancer Institute of Japanese Foundation for Cancer Research, 3-8-31 Ariake, Koto-ku, Tokyo 135-8550, Japan. [4]Graduate School of Frontier Biosciences, Osaka University, Suita, Osaka 565-0871, Japan. [5]Cell Biology Center, Institute of Innovative Research, Tokyo Institute of Technology, 4259 Nagatsuta-cho, Midori-ku, Yokohama 226-8501, Japan.

[6] These authors equally contributed to this work.

Correspondence should be addressed to H.K. (e-mail: kurumizaka@iam.u-tokyo.ac.jp)

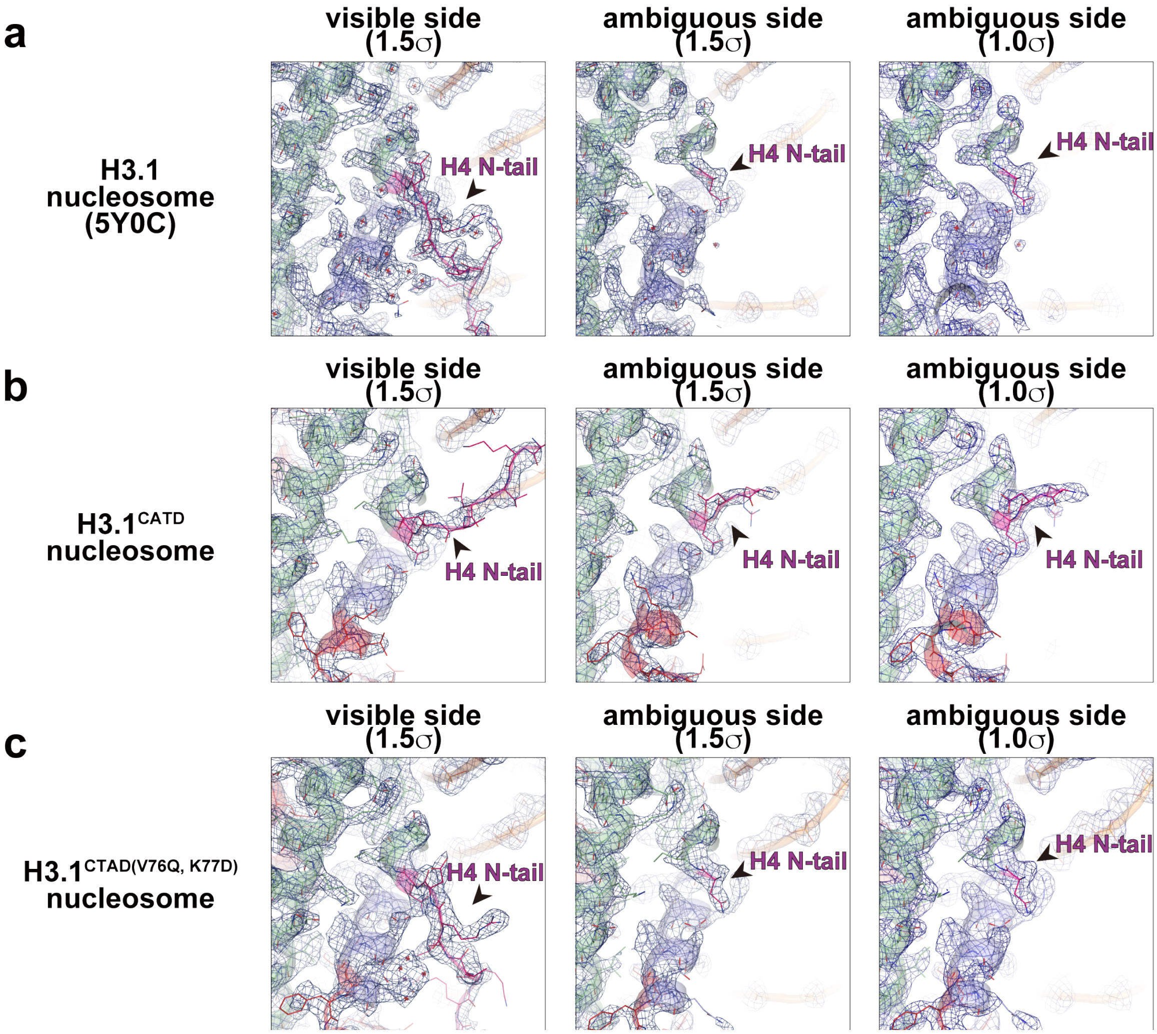

Supplementary Figure 1. Electron density maps around the H4 N-terminal tail in the H3.1 nucleosome, H3.1^{CATD} nucleosome, and H3.1$^{CATD(V76Q, K77D)}$ nucleosome. **a-c,** The electron density maps around the H4 N-terminal tails in the H3.1 nucleosome (a), H3.1^{CATD} nucleosome (b), and H3.1$^{CATD(V76Q, K77D)}$ nucleosome (c). The blue mesh shows the 2m*F*o-D*F*c map, which was calculated and contoured at the 1.0σ or 1.5σ level. The regions derived from H3.1, CENP-A, H4 N-terminal tail (amino acid residues 1-25), and H4 core region (amino acid residues 26-102) are colored blue, red, pink, and green, respectively.

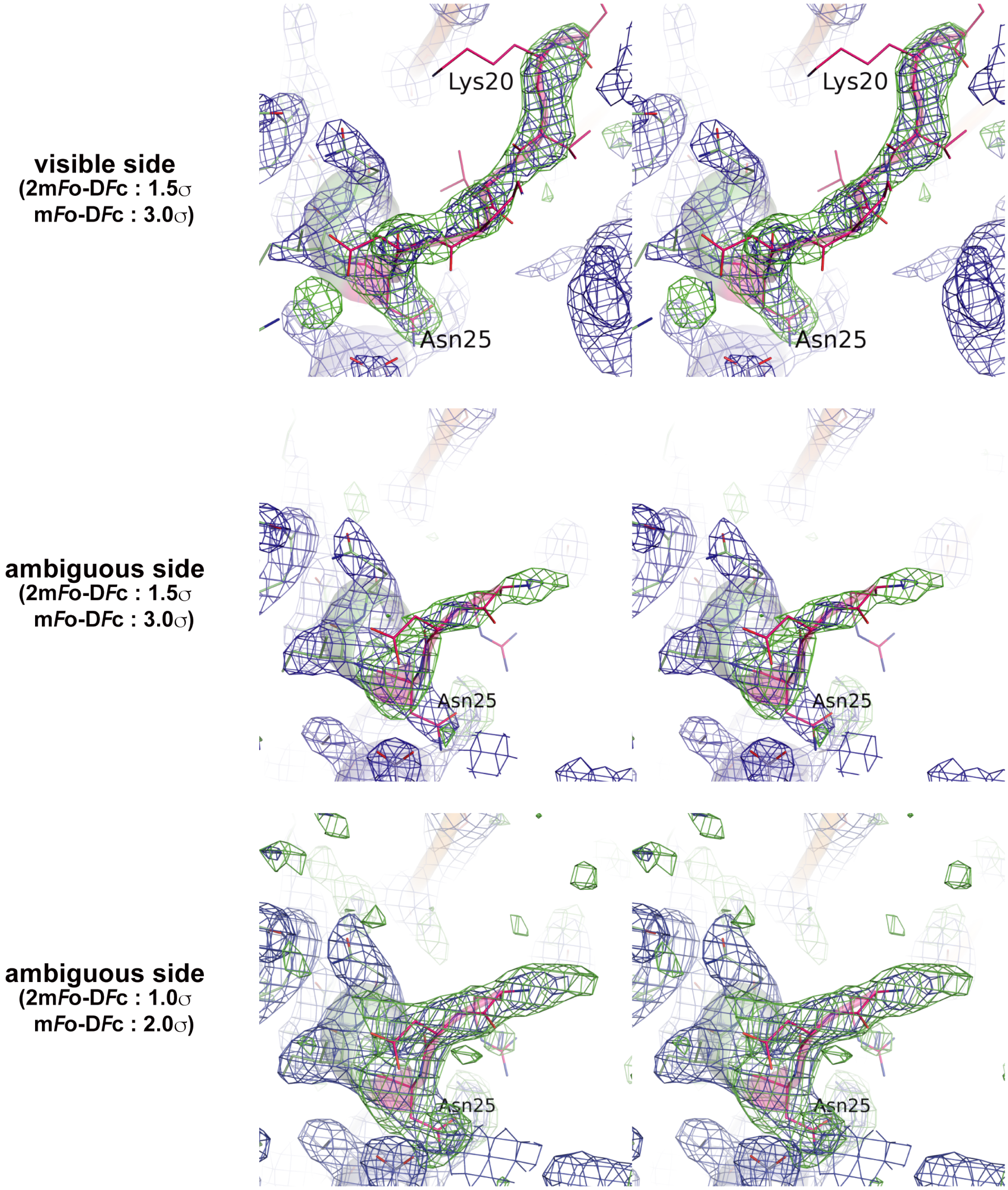

Supplementary Figure 2. The omit map around the H4 N-terminal tail in the H3.1^{CATD} nucleosome.
Stereoviews of the electron density map around the H4 N-terminal tail in the H3.1^{CATD} nucleosome. The electron density map was calculated using the modified atomic coordinates, in which amino acid residues 1-25 of H4 were deleted from the H3.1^{CATD} nucleosome. The 2m*F*o-D*F*c map was contoured at the 1.0σ or 1.5σ level and shown by a blue mesh. The m*F*o-D*F*c map was contoured at the 2.0σ or 3.0σ level and shown by a green mesh. The regions derived from H4 N-terminal tail (amino acid residues 1-25), and H4 core region (amino acid residues 26-102) are colored pink, and green, respectively.

a

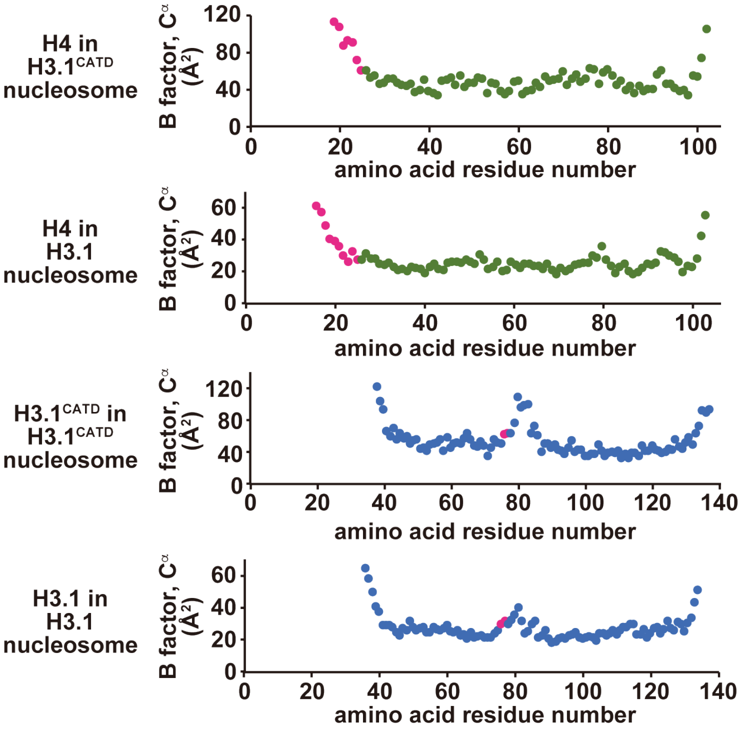

b

H3.1 nucleosome (5Y0C)

ion	B factor (Å²)
Mn (H3.1D77-**Mn²⁺**-H₂O-H4L22)	27.1
O (H3.1D77-Mn²⁺-H₂**O**-H4L22)	21.2
O (H3.1Q76-H₂**O**-H4L22)	25.4

Supplementary Figure 3. B factors of the H3.1, H3.1^{CATD}, and H4 molecules in the nucleosome structures.

a, The B factors of the Cα atoms of the H4 molecule (ambiguous side) and the H3.1 or H3.1^{CATD} molecule in the crystal structures of the H3.1 nucleosome (5Y0C) and the H3.1^{CATD} nucleosome are plotted against the amino acid residues. Pink dots indicate the amino acid residues involved in the interaction between the H4 N-terminal tail and the H3 residues 76 and 77. **b,** The B factors of the Mn^{2+} ion and oxygen atoms of water molecules, which mediate the interaction between the H4 N-terminal tail and H3.1, are presented in the table. The corresponding Mn^{2+} ion and oxygen atoms are indicated in red.

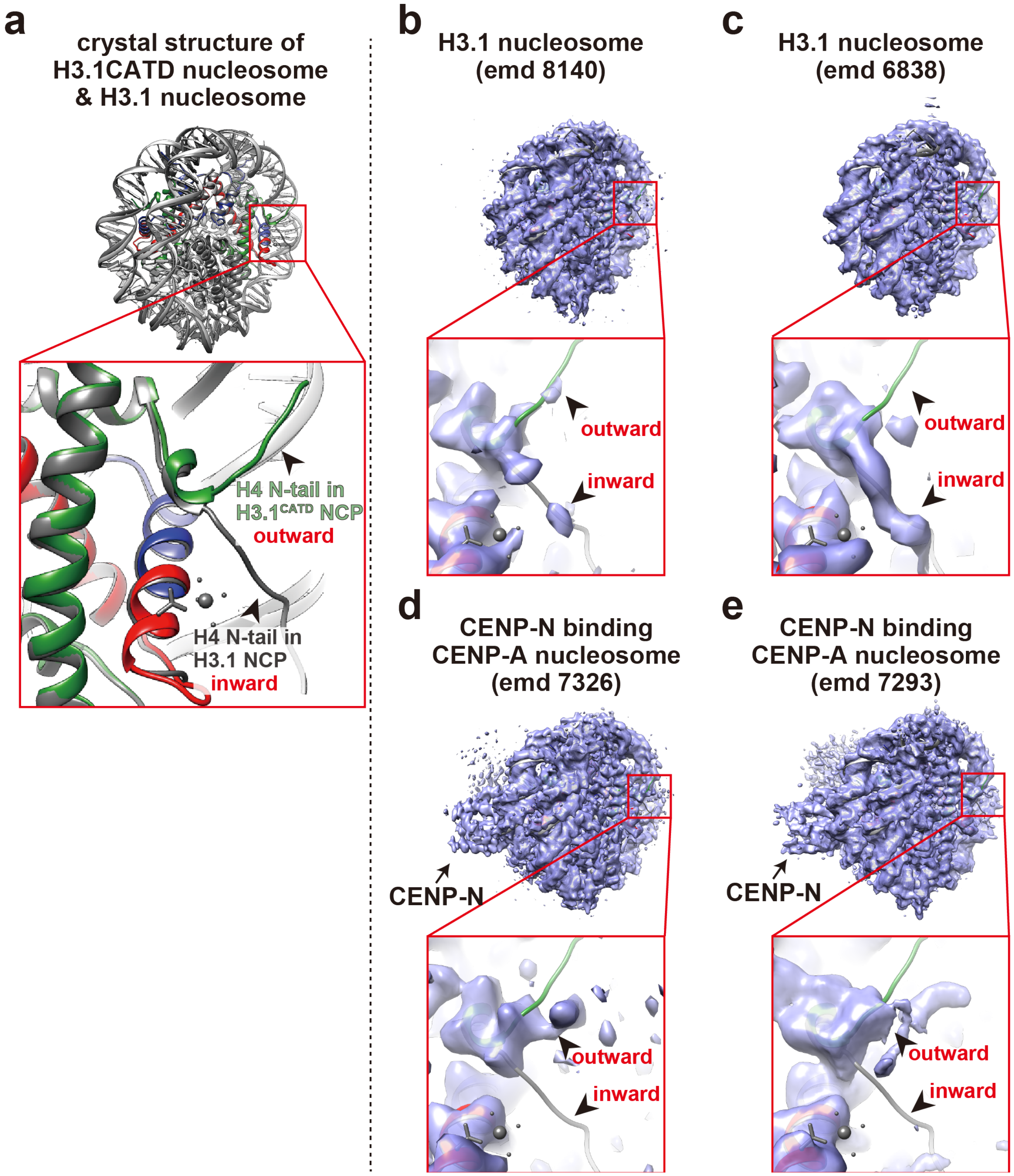

Supplementary Figure 4. The EM density maps around the H4 N-terminal tail in the nucleosome structure, revealed by the previously reported cryo-EM studies. **a,** Structural comparison of the H4 N-terminal tails between the H3.1^{CATD} nucleosome and the H3.1 nucleosome (PDB ID: 5Y0C), viewed from the same angle as in Supplementary Figure 4b-e. **b and c,** The EM density maps around the H4 N-terminal tail in the H3.1 nucleosome (emd 8140 (b) and emd 6838 (c)). **d and e,** The EM density maps of the CENP-A nucleosome complexed with CENP-N (emd 7326 (d) and emd 7293 (e)) around the H4 N-terminal tail in the non-CENP-N binding side.

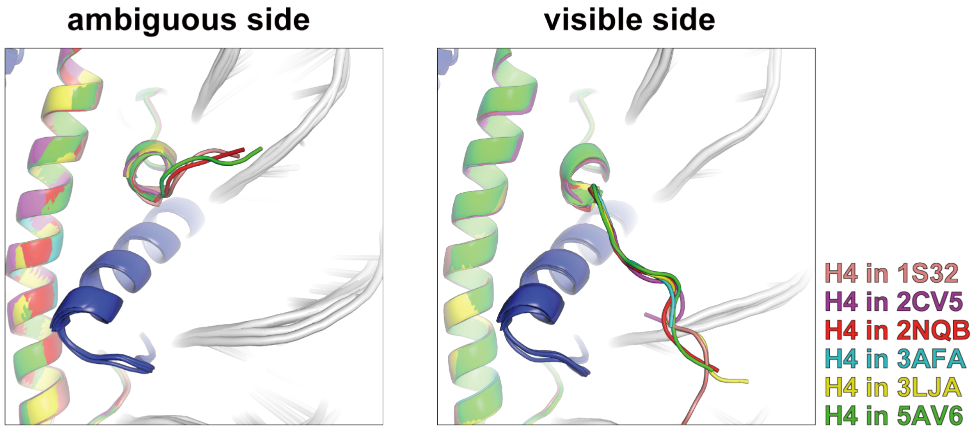

Supplementary Figure 5. Close-up views of the H4 N-terminal tails in the H3.1 nucleosome structure, revealed by the previously reported X-ray crystallography. Comparison of the H4 N-terminal tail structures in the H3.1 nucleosomes. The H4 structures in the human H3.1 nucleosomes with PDB IDs 2CV5, 3AFA, and 5AV6 are colored pink, blue, and green, respectively. The H4 structures in the *Xenopus laevis* H3.1 nucleosomes with PDB IDs 3LJA and 1S32 are colored yellow and orange. The H4 structure in the *Drosophila melanogaster* H3.1 nucleosome, with PDB ID 2NQB, is colored red.

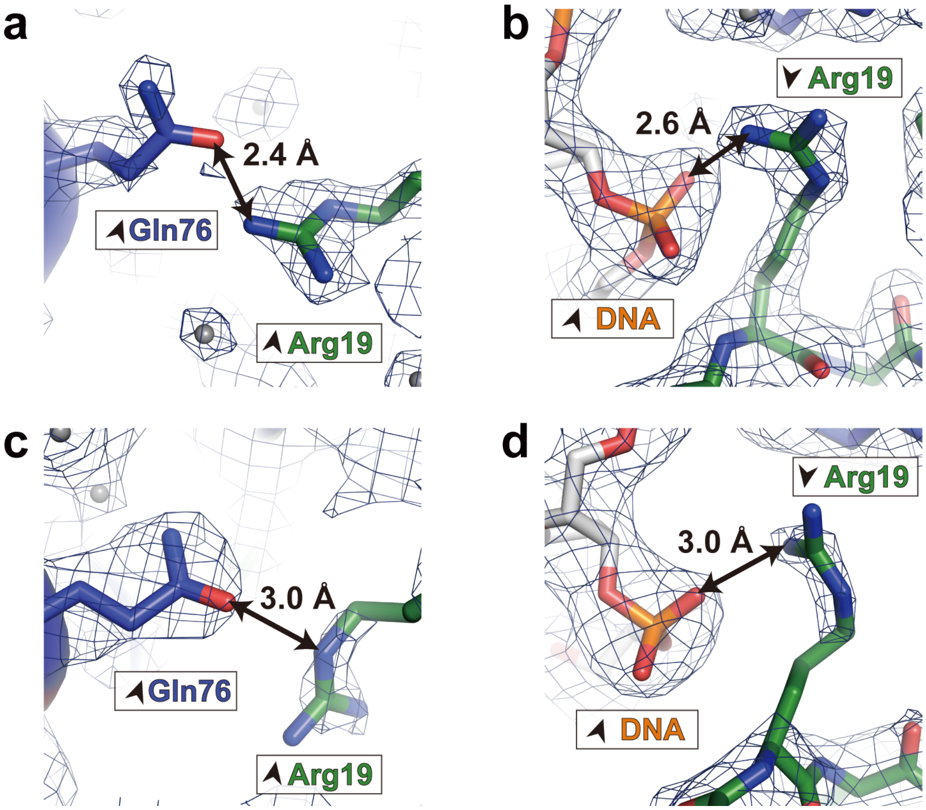

Supplementary Figure 6. Distances between the H4 Arg19 and H3 Gln76 residues or the nearest DNA backbone phosphate in the H3.1 and H3.1CATD$^{(V76Q, K77D)}$ nucleosomes.

a, The black arrow indicates the distance between the H4 Arg19 and H3.1 Gln76 residues in the H3.1 nucleosome. **b,** The black arrow indicates the distance between the H4 Arg19 residue and the nearest DNA backbone phosphate in the H3.1 nucleosome. **c,** The black arrow indicates the distance between the H4 Arg19 and H3.1 Gln76 residues in the H3.1CATD$^{(V76Q, K77D)}$ nucleosome. **d,** The black arrow indicates the distance between the H4 Arg19 residue and the nearest DNA backbone phosphate in the H3.1CATD$^{(V76Q, K77D)}$ nucleosome. The gray spheres represent the oxygen atoms of water molecules. The blue mesh shows the 2m*F*o-D*F*c map, which was calculated and contoured at the 1.0σ level.

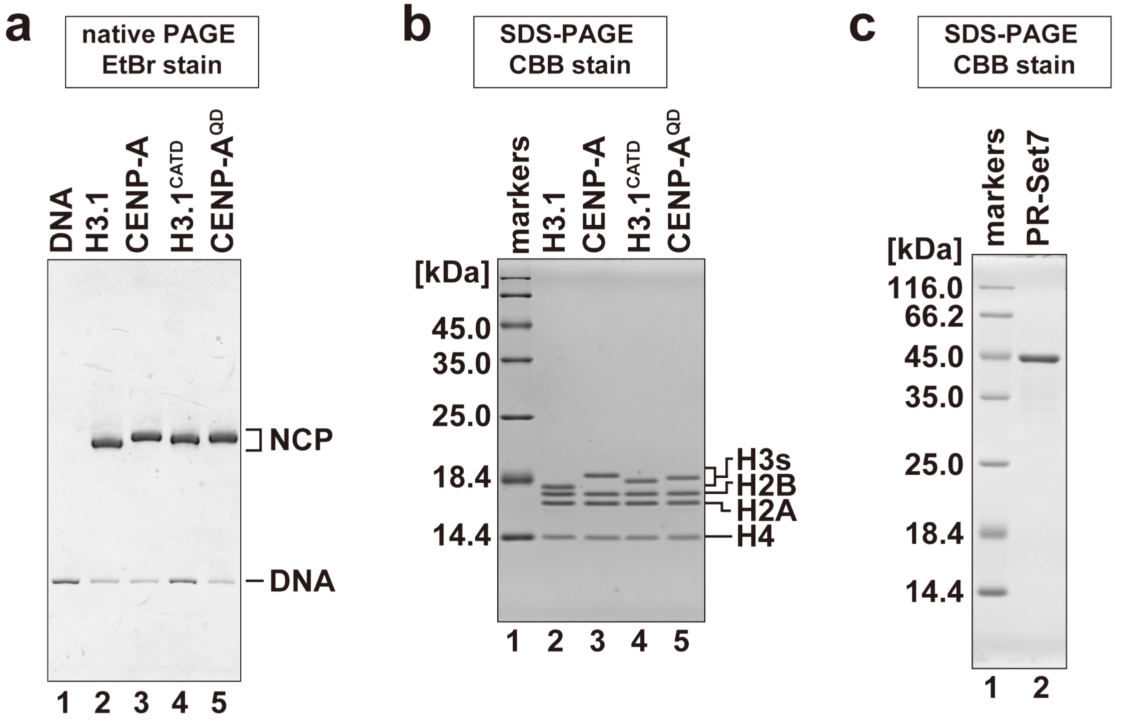

Supplementary Figure 7. Purified nucleosomes and PR-Set7 used in the *in vitro* methyltransferase assay.

a, Purified nucleosomes were analyzed by native PAGE. Lane 1 indicates the 156 base-pair Widom601 DNA. Lanes 2, 3, 4, and 5 indicate the nucleosomes containing H3.1, CENP-A, H3.1^{CATD}, and CENP-A^{QD}. **b**, Histone contents of purified nucleosomes were analyzed by SDS-PAGE. Lane 1 indicates molecular mass markers. Lanes 2, 3, 4, and 5 indicate the nucleosomes containing H3.1, CENP-A, H3.1^{CATD}, and CENP-A^{QD}. **c**, SDS-PAGE analysis of purified His$_6$-tagged PR-Set7. Lane 1 indicates molecular mass markers. Lane 2 indicates purified His$_6$-tagged PR-Set7.

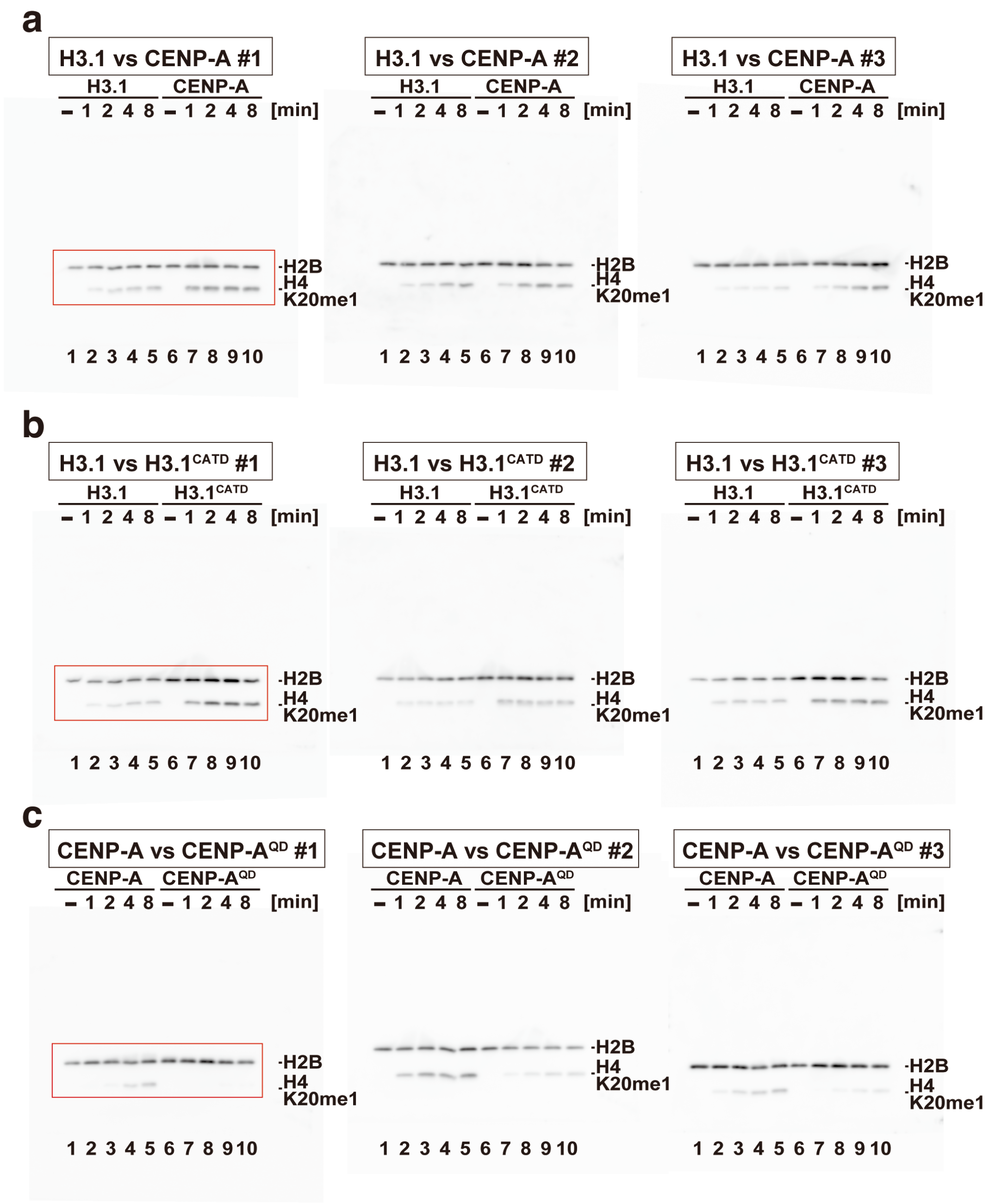

Supplementary Figure 8. Replicated experiments of the *in vitro* methyltransferase assay.

The full images of the replicated experiments of the *in vitro* methyltransferase assays shown in Figure 3d, e, and f. **a**, Lanes 1-5 and 6-10 indicate results for the H3.1 and CENP-A nucleosomes, respectively. The nucleosomes (0.48 µM) were incubated with 0.30 µM His_6-tagged PR-Set7. **b**, Lanes 1-5 and 6-10 indicate results for the H3.1 and H3.1^{CATD} nucleosomes, respectively. The nucleosomes (0.48 µM) were incubated with 0.30 µM His_6-tagged PR-Set7. **c**, Lanes 1-5 and 6-10 indicate results for the CENP-A and CENP-A^{QD} nucleosomes, respectively. The nucleosomes (0.48 µM) were incubated with 0.15 µM His_6-tagged PR-Set7. The red rectangles show the representative images used for Figure 3d, e, and f.

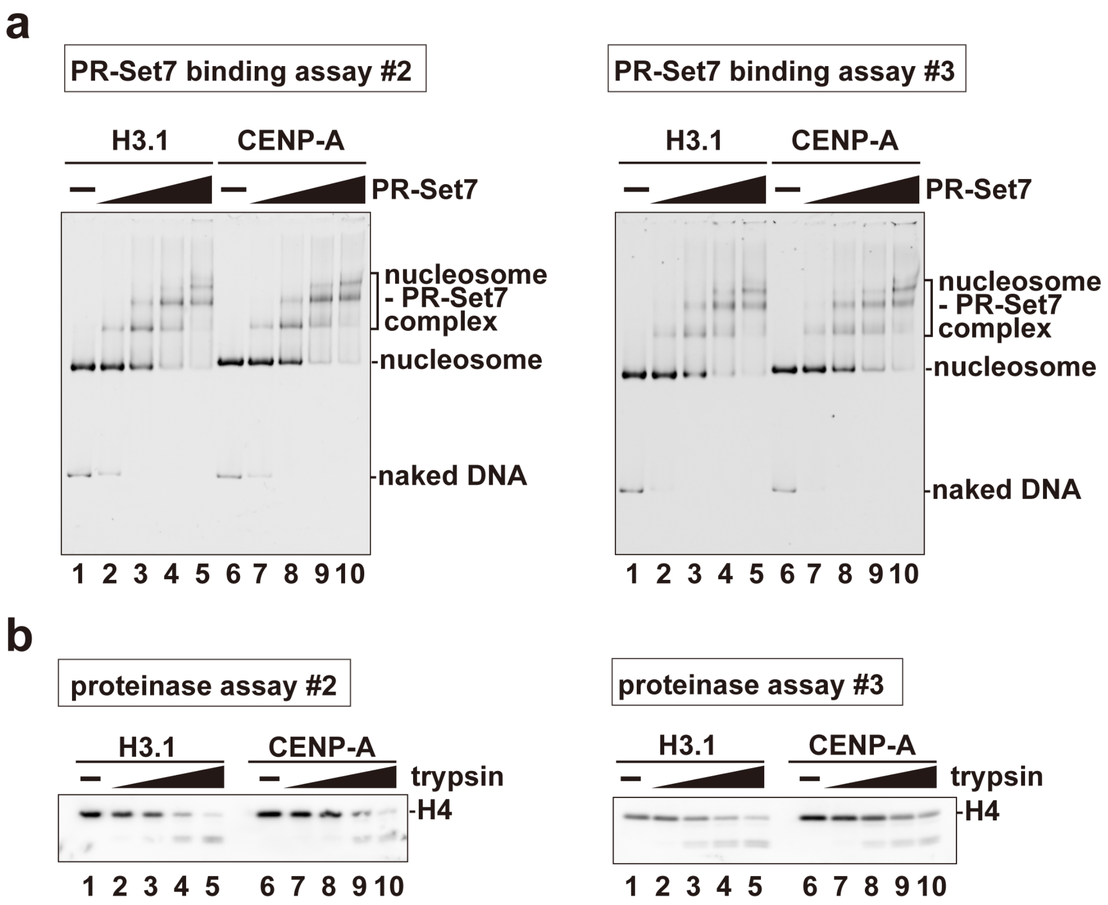

Supplementary Figure 9. Replicated experiments of Fig. 3g and h.
The PR-Set7 gel shift assay and the proteinase assay with trypsin were independently performed three times. **a**, The gel shift assay with His_6-tagged PR-Set7. Lanes 1-5 and 6-10 indicate results for the H3.1 and CENP-A nucleosomes, respectively. **b**, Proteinase assay. Lanes 1-5 and 6-10 indicate results for the H3.1 and CENP-A nucleosomes, respectively.

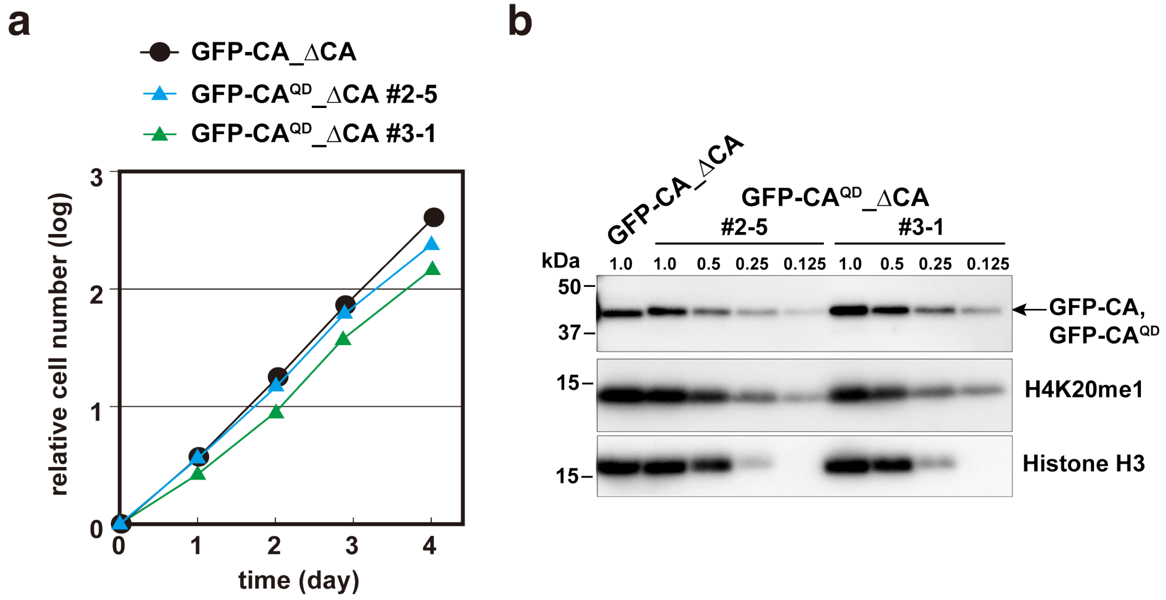

Supplementary Figure 10. CENP-A-deficient cells expressing either GFP-tagged CENP-A or CENP-A^{QD}.

a, Growth curves of CENP-A-deficient cell lines expressing either GFP-tagged CENP-A (GFP-CA_ΔCA) or CENP-A^{QD} (GFP-CA^{QD}_ΔCA). For GFP-CA^{QD}_ΔCA, two independent cell lines (#2-5, #3-1) were used. **b,** Immunoblot analyses with anti-GFP (Upper panel), anti-H4K20me1 (Middle panel), and anti-H3 (Lower panel) antibodies, using the chromatin fraction prepared from CENP-A-deficient cells expressing either GFP-tagged CENP-A (GFP-CA_ΔCA) or CENP-A^{QD} (GFP-CA^{QD}_ΔCA: #2-5 and #3-1 lines). Dilution series (1.0, 0.5, 0.25, 0.125) of the protein samples from the GFP-CA^{QD}_ΔCA cells (#2-5 and #3-1) were used to compare the expression level of each protein. An immunoblot with anti-H3 was used as a control (Lower panel). The expression level of GFP-tagged CENP-A^{QD} in #2-5 cells was comparable to that of GFP-tagged CENP-A in GFP-CA_ΔCA cells, while that of GFP-tagged CENP-A^{QD} was twice as high in #3-1 cells as compared to the GFP-CENP-A expression in GFP-CA_ΔCA cells. The modification levels of H4K20me1 in bulk chromatin were similar in all cell lines.

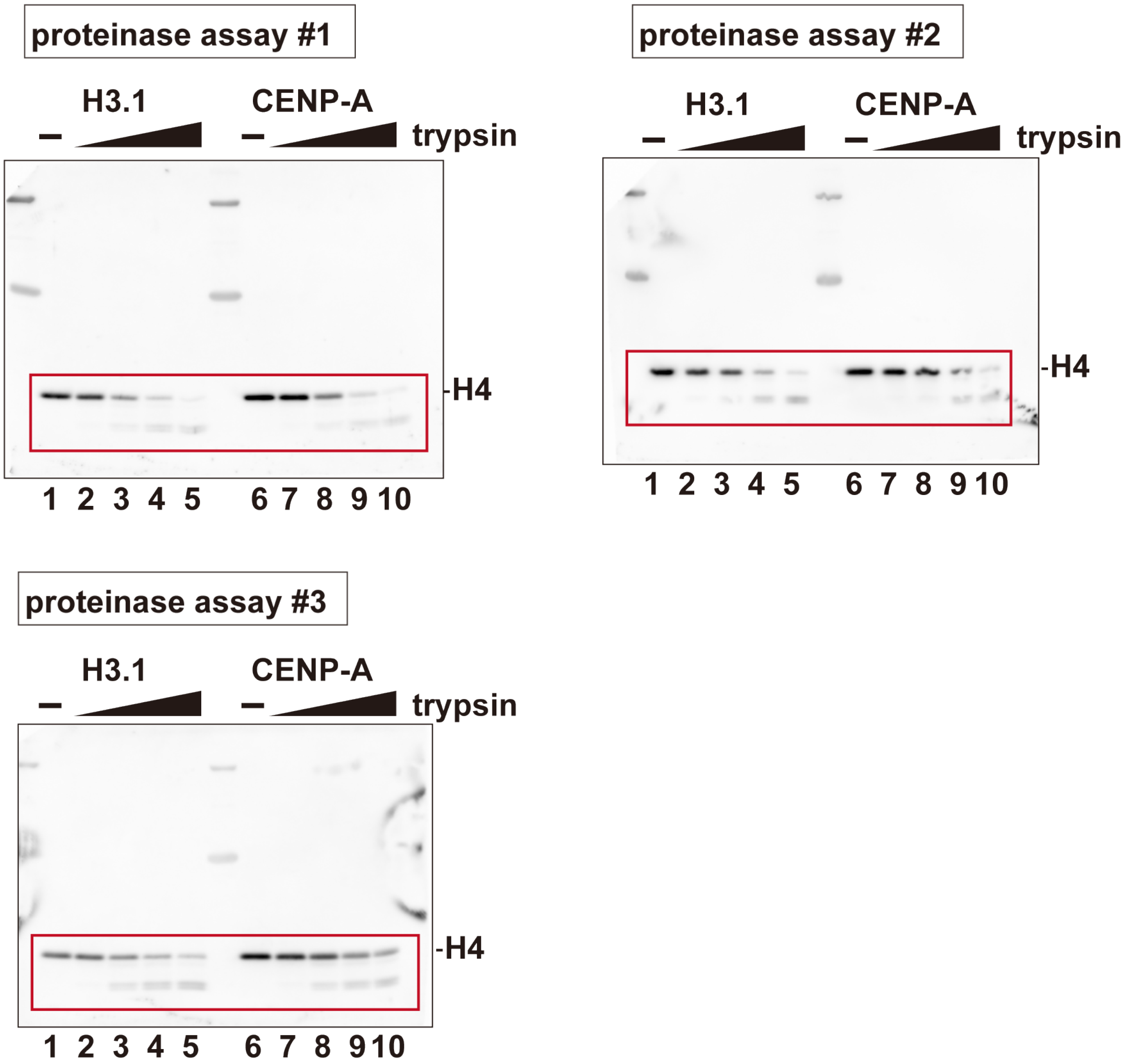

Supplementary Figure 11 Full images of the western blots shown in Fig. 3g and Supplementary Figure 9b. The proteinase assay was independently performed three times. The red rectangles show the representative images used for Fig. 3g and Supplementary Figure 9b.

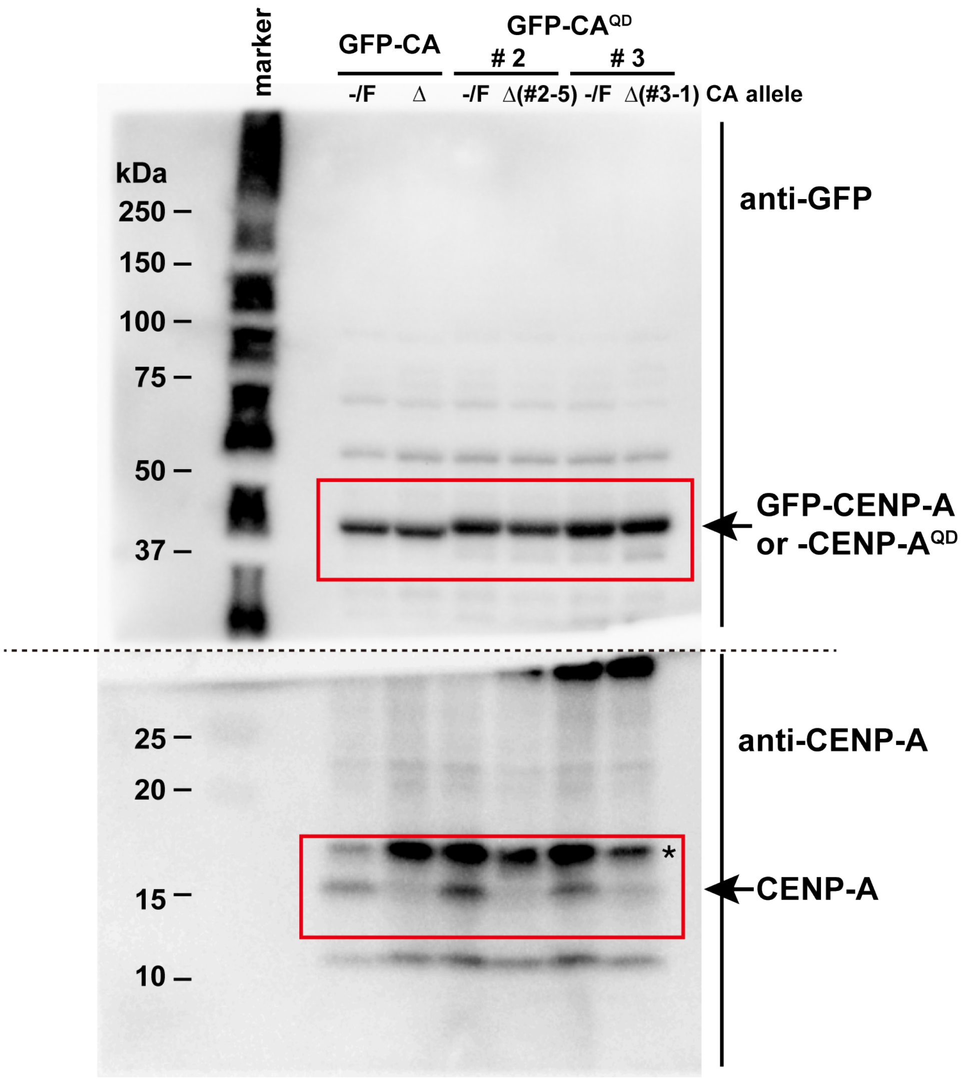

Supplementary Figure 12. Full image of the immunoblot shown in Fig. 4c. Immunoblot samples were prepared from cells expressing either GFP-tagged CENP-A (GFP-CA) or CENP-A^{QD} (GFP-CA^{QD}), before (-/F) and after (Δ) the endogenous CENP-A knockout. All samples were blotted on the same membrane. The membrane was then cut at the indicated dashed line and used for different antibodies. Expression of GFP-tagged CENP-A and CENP-A^{QD} and depletion of the endogenous CENP-A were confirmed by immunoblot analyses with an anti-GFP antibody (upper part of the membrane) and an anti-CENP-A antibody (lower part of the membrane), respectively. The asterisk indicates nonspecific bands. The regions enclosed by red rectangles were used for Fig. 4c.

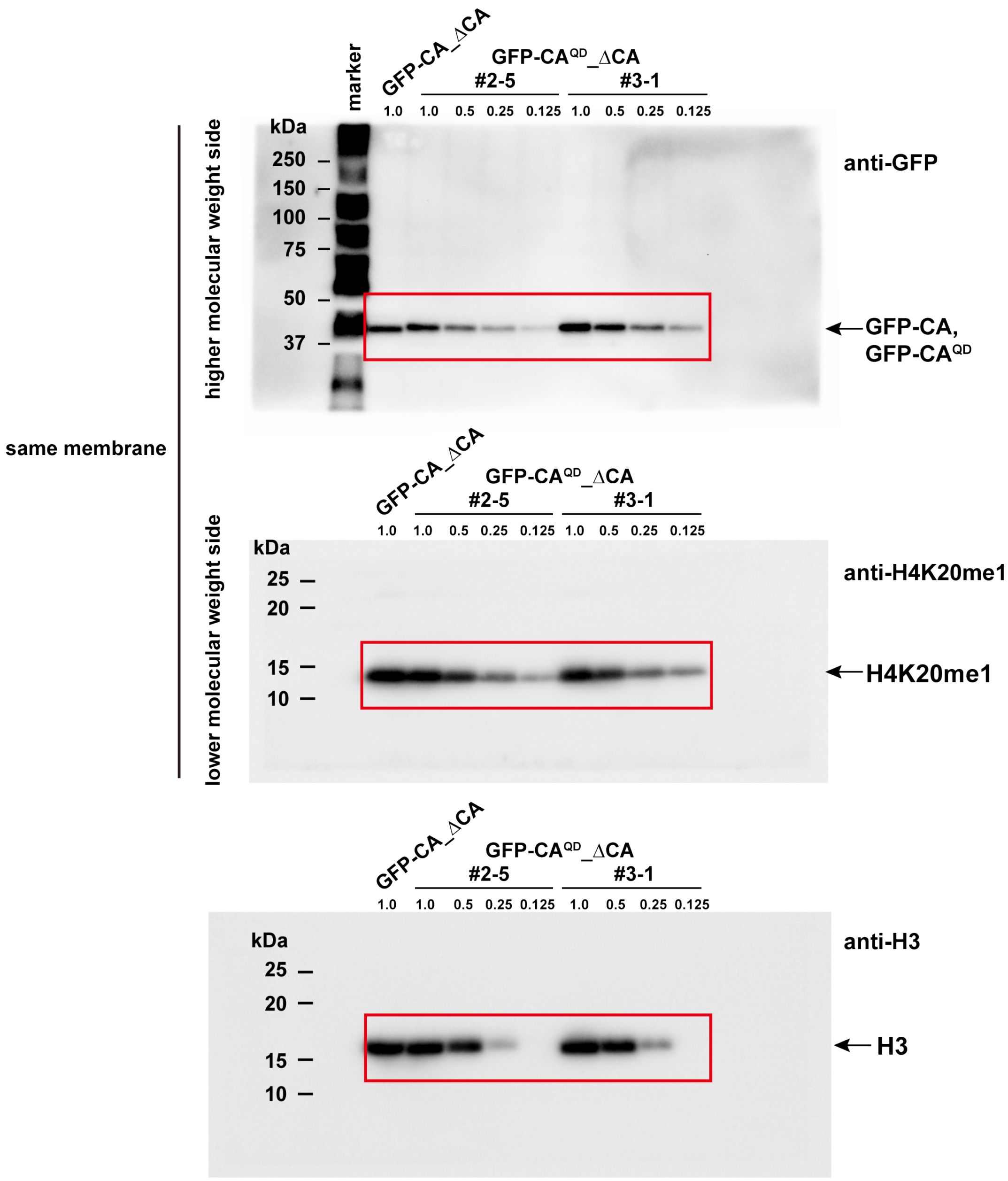

Supplementary Figure 13. Full image of the immunoblot shown in Supplementary Figure 10b.

Immunoblot samples were prepared from CENP-A-deficient cells expressing either GFP-tagged CENP-A (GFP-CA_ΔCA) or CENP-A^{QD} (GFP-CA^{QD}_ΔCA, #2-5 and #3-1). The samples from GFP-CA_ΔCA and the dilution series (1.0, 0.5, 0.25, 0.125) from GFP-CA^{QD}_ΔCA (#2-5, #3-1) were blotted on the same membrane. The membrane was then cut, and the higher and lower molecular weight regions were used for immunoblot analyses with anti-GFP and anti-H4K20me1, respectively. For the immunoblot with the anti-H3 antibody, 10-fold reduced amounts of all samples were blotted on the same membrane, in the same order as for the immunoblots with anti-GFP or anti-H4K20me1. The membrane was then cut, and the lower molecular weight side was blotted. The areas enclosed by red rectangles were used for Supplementary Fig. 10b.

Supplementary Table 1. X-ray crystallography data collection and refinement statistics

	H3.1^{CATD} nucleosome	H3.1$^{CATD(V76Q, K77D)}$ nucleosome
**Data collection**		
Space group	$P2_12_12_1$	$P2_12_12_1$
Cell dimensions		
a, *b*, *c* (Å)	99.641, 100.745, 173.431	99.54, 109.00, 170.14
α, β, γ (°)	90.000, 90.000, 90.000	90.000, 90.000, 90.000
Resolution (Å)	50.00-2.71 (2.81-2.71)	50-2.58 (2.73-2.58)
R_{merge}	6.8 (46.6)	14.5 (160.9)
$I / \sigma I$	19.3 (2.8)	14.72 (1.68)
Completeness (%)	96.5 (90.3)	99.6 (98.3)
$CC_{1/2}$ in outer shell	0.797	0.707
Redundancy	5.7 (4.8)	13.2 (13.6)
**Refinement**		
Resolution (Å)	47.884 - 2.730	49.276 - 2.58
No. reflections	45284	58890
R_{work} / R_{free}	20.29/26.14	21.33/24.93
No. atoms		
Protein	6028	6024
DNA	5980	5980
Water	0	4
Ion	0	10
B-factors		
Protein	58.1	56.1
DNA	108.0	86.5
Water	-	42.9
Ion	-	76.2
R.m.s. deviations		
Bond lengths (Å)	0.010	0.008
Bond angles (°)	1.208	1.026

* The crystal structures of the H3.1^{CATD} nucleosome and H3.1$^{CATD(V76Q, K77D)}$ nucleosome were determined from single crystals.

*Values in parentheses are for highest-resolution shell.

Supplementary Table 2. Primers used in this study

For pGEM-T-easy-156 base-pair DNA preparation, top	AGGATCCGATATCCCCTTTGAATTCGTACGTGCG TTTAAGCGGTGCTAG
For pGEM-T-easy-156 base-pair DNA preparation, bottom	AGGTACCAAGATCTGATATCAAATTGAATCCAGG ATCGACAATCCCGGTGCCGA
For pHCE-H3.1^{CATD} preparation, H3 amplification top	GTACCATGCTAAGCGAGTGACTATTATGCCC
For pHCE-H3.1^{CATD} preparation, H3 amplification bottom	CACCAGGCGCTGGAACGGCAGCTTCC
For pHCE-H3.1^{CATD} preparation, CATD amplification top	CGTGAAATTTGCGTTAAATTTACGCGTG
For pHCE-H3.1^{CATD} preparation, CATD amplification bottom	CAGGGTTAACAGATACGCATCTTCAAACAGAT
For pHCE-H3.1^{CATD} preparation, fix mutation 1 top	CCAGCGCCTGGTGCGTGAAATTTG
For pHCE-H3.1^{CATD} preparation, fix mutation 1 bottom	CAAATTTCACGCACCAGGCGCTGG
For pHCE-H3.1^{CATD} preparation, fix mutation 2 top	TCTGTTAACCCTGCATGCTAAGCGAGTGA
For pHCE-H3.1^{CATD} preparation, fix mutation 2 bottom	TCACTCGCTTAGCATGCAGGGTTAACAGA
For pET15b-H2A$^{L51M, L58M, L93M}$ preparation, L51M top	GCGCGCCGGTGTATATGGCGGCGGTGCTTG
For pET15b-H2A$^{L51M, L58M, L93M}$ preparation, L51M bottom	CAAGCACCGCCGCCATATACACCGGCGCGC
For pET15b-H2A$^{L51M, L58M, L93M}$ preparation, L58M top	CGGTGCTTGAGTACATGACCGCCGAGATCC
For pET15b-H2A$^{L51M, L58M, L93M}$ preparation, L58M bottom	GGATCTCGGCGGTCATGTACTCAAGCACCG
For pET15b-H2A$^{L51M, L58M, L93M}$ preparation, L93M top	AATGACGAGGAGATGAATAAACTTTTGGGG
For pET15b-H2A$^{L51M, L58M, L93M}$ preparation, L93M bottom	CCCCAAAAGTTTATTCATCTCCTCGTCATT
For pET15b- CENP-A^{QD} preparation, mutagenesis top	CAAGACTTTACCCGCGGCGTGGATTTTAACTGGC A
For pET15b- CENP-A^{QD} preparation, mutagenesis bottom	GCAAATTTCGCGCGCCAGGCGGCTAAACGGCAG TTT
For pHCE-H3.1$^{CATD(V76Q, K77D)}$ preparation, mutagenesis top	GACTTTACGCGTGGTGTGGATTTTAACTGGCAGG CACAGG
For pHCE-H3.1$^{CATD(V76Q, K77D)}$ preparation, mutagenesis bottom	TTGGCAAATTTCACGCACCAGGCGCTGGAACGGC AGCTTC
For pET15b-PRSet7 preparation, PRSet7 amplification top	TTCCAGGGGCCCCATATGGCTAGAGGCAGGAAG ATGTCCA
For pET15b-PRSet7 preparation, PRSet7 amplification bottom	GCAGCCGGATCCTTAATGCTTCAGCCACGGGTGG GCTTCA
For pET15b-PRSet7 preparation, pET15b amplification top	TAAGGATCCGGCTGCTAACAAAGCC
For pET15b-PRSet7 preparation, pET15b amplification bottom	ATGGGGCCCCTGGAACAGAACTTCC
