# Supplementary material for: The CENP-A centromere targeting domain facilitates H4K20 monomethylation in the nucleosome by structural polymorphism
Source: Nat Commun. 2019 Feb 4;10:576. doi: 10.1038/s41467-019-08314-x (PMC6362020; doi:10.1038/s41467-019-08314-x)
Supplement: Supplementary file 3 — Reporting Summary [file 41467_2019_8314_MOESM3_ESM.pdf]

nature research

Corresponding author(s): Hitoshi Kurumizaka

Reporting Summary

Nature Research wishes to improve the reproducibility of the work that we publish. This form provides structure for consistency and transparency in reporting. For further information on Nature Research policies, see Authors & Referees and the Editorial Policy Checklist.

Statistical parameters

When statistical analyses are reported, confirm that the following items are present in the relevant location (e.g. figure legend, table legend, main text, or Methods section).

n/a	Confirmed	
☐	☒	The exact sample size (*n*) for each experimental group/condition, given as a discrete number and unit of measurement
☐	☒	An indication of whether measurements were taken from distinct samples or whether the same sample was measured repeatedly
☒	☐	The statistical test(s) used AND whether they are one- or two-sided<br>*Only common tests should be described solely by name; describe more complex techniques in the Methods section.*
☒	☐	A description of all covariates tested
☒	☐	A description of any assumptions or corrections, such as tests of normality and adjustment for multiple comparisons
☒	☐	A full description of the statistics including central tendency (e.g. means) or other basic estimates (e.g. regression coefficient) AND variation (e.g. standard deviation) or associated estimates of uncertainty (e.g. confidence intervals)
☒	☐	For null hypothesis testing, the test statistic (e.g. *F*, *t*, *r*) with confidence intervals, effect sizes, degrees of freedom and *P* value noted<br>*Give P values as exact values whenever suitable.*
☒	☐	For Bayesian analysis, information on the choice of priors and Markov chain Monte Carlo settings
☒	☐	For hierarchical and complex designs, identification of the appropriate level for tests and full reporting of outcomes
☒	☐	Estimates of effect sizes (e.g. Cohen's *d*, Pearson's *r*), indicating how they were calculated
☒	☐	Clearly defined error bars<br>*State explicitly what error bars represent (e.g. SD, SE, CI)*

Our web collection on statistics for biologists may be useful.

Software and code

Policy information about availability of computer code

Data collection

For the sturctural analyses, the diffraction data were collected at the BL41XU station of SPring-8, Harima, Japan.

For the genomic analyses, ChIP-seq libraries were sequenced using an Illumina HiSeq 2500 (100 bp single end sequencing).

Data analysis

For the sturctural analysis of H3.1CATD nucleosome, the diffraction data were integrated and scaled with the HKL2000 program and the CCP4 program suite. For the H3.1 CATD(V76Q, K77D) nucleosome, the diffraction data were subjected to integration, scaling, and FreeR flag generation with the XDS program suite. The resolution limit was determined with the Aimless software. The crystal structures were determined by molecular replacement with the Phaser program, using the human canonical nucleosome structures (PDB ID: 3AFA for the H3.1CATD nucleosome, and modified 2CV5, in which the atomic coordinates of histone H3 were removed, for the H3.1CATD(V76Q, K77D) nucleosome) as the search models. The structural models were refined using the Phenix program suite and the Coot program. For the H3.1CATD nucleosome, 98.65%, 1.35%, and 0% of the amino acids were assigned in the Ramachandran favored, allowed, and outlier regions, respectively. For the H3.1CATD(V76Q, K77D) nucleosome, 97.56%, 2.44%, and 0% of the amino acids were assigned in the Ramachandran favored, allowed, and outlier regions, respectively. To calculate an unbiased Fo-Fc omit map, the atomic coordinates for the H4 1-25 residues were deleted from the H3.1CATD nucleosome structure. With this model structure, the Fo-Fc omit map was calculated by the Phenix program. Structural graphics were displayed using the PyMOL program (http://pymol.org) or Chimera program.

For the genomic analyses, the sequence data were mapped to the Chicken Genome database (NCBI, Gallus_gallus-4.0), as well as to the Drosophila genome database (NCBI, Drosophila-dm6), with the Burrows-Wheeler Aligner version 0.6.2 mapping tool. Correction factors for ChIP-seq with anti-H4K20me1 or with anti-GFP were generated independently, using the numbers of sequence-reads mapped on the

Drosophila genome. The numbers of sequence-reads mapped on the Chicken genome were then normalized, using the correction factors.

For manuscripts utilizing custom algorithms or software that are central to the research but not yet described in published literature, software must be made available to editors/reviewers upon request. We strongly encourage code deposition in a community repository (e.g. GitHub). See the Nature Research guidelines for submitting code & software for further information.

Data

Policy information about availability of data

All manuscripts must include a data availability statement. This statement should provide the following information, where applicable:

- Accession codes, unique identifiers, or web links for publicly available datasets
- A list of figures that have associated raw data
- A description of any restrictions on data availability

The raw full image data of Figure 3d, e, and f were shown in Extended Data Figure 8. The raw full image data of Fig. 3g and Extended Data Figure 9 were shown in Extended Data Figure 11. The raw full image data of Fig. 4c and Extended Data Figure 10b were shown in Extended Data Figure 12 and Extended Data Figure 13, respectively.
The coordinates and structure factors of the H3.1CATD nucleosome and the H3.1CATD(V76Q, K77D) nucleosome have been deposited in the Protein Data Bank, under the accession codes 5Z23 and 5ZBX, respectively. Spike-in ChIP-seq data with anti-H4K20 me1 or anti-GFP antibodies were submitted to the DDBJ Sequence Read Archive with the following accession number: DRA007099.

Field-specific reporting

Please select the best fit for your research. If you are not sure, read the appropriate sections before making your selection.

☒ Life sciences ☐ Behavioural & social sciences ☐ Ecological, evolutionary & environmental sciences

For a reference copy of the document with all sections, see nature.com/authors/policies/ReportingSummary-flat.pdf

Life sciences study design

All studies must disclose on these points even when the disclosure is negative.

Sample size	Since our study did not contain statistical test, we did not perform sample-size calculation.
Data exclusions	No data were excluded from analyses.
Replication	The reproducibility for all biochemical analyses was confirmed by three independent experiments. The reproducibility for genomic analysis was confirmed by two independent experiments.
Randomization	This was not relevant to our study.
Blinding	This was not relevant to our study.

Reporting for specific materials, systems and methods

Materials & experimental systems

n/a	Involved in the study	
☒	☐	Unique biological materials
☐	☒	Antibodies
☐	☒	Eukaryotic cell lines
☒	☐	Palaeontology
☒	☐	Animals and other organisms
☒	☐	Human research participants

Methods

n/a	Involved in the study	
☐	☒	ChIP-seq
☒	☐	Flow cytometry
☒	☐	MRI-based neuroimaging

Antibodies

Antibodies used	mouse anti-H4K20 monomethylation (#CMA421, Hayashi-Takanaka, Y. et al. Chromosome Res. 23, 753-766 (2015)) anti-H2B monoclonal antibody (53H3: Cell Signaling, https://www.cellsignal.com/products/primary-antibodies/histone-h2b-53h3-mouse-mab/2934),

anti-H4 rabbit polyclonal antibody (Abcam, ab7311, https://www.abcam.com/histone-h4-antibody-chip-grade-ab7311.html),
Amersham ECL mouse IgG, HRP-linked F(ab')$_2$ fragment from sheep (NA9310: GE Healthcare, https://www.sigmaaldrich.com/catalog/product/sigma/gena93101ml?lang=en&region=US),
HRP-anti rabbit-IgG F(ab')$_2$ fragment (GE Healthcare, NA9340, https://www.sigmaaldrich.com/catalog/product/sigma/gena93401ml?lang=en&region=US),
rabbit anti-GFP (MBL, https://www.mblintl.com/products/598),
rabbit anti-chicken CENP-A (Regnier, V. et al. Mol. Cell. Biol. 25, 3967-3981 (2005)
Rat anti-pan H3 (#140-1G1, Nozawa, R.S., et al. Nat. Cell. Biol. 12, 719-727 (2010).)
HRP-conjugated anti-rabbit IgG (Jackson ImmunoResearch, https://www.jacksonimmuno.com/catalog/products/111-035-144),
HRP-conjugated anti-mouse IgG (Jackson ImmunoResearch, https://www.jacksonimmuno.com/catalog/products/115-035-003)

Validation

The commercial antibodies were validated by their producers. The antibodies provided by the collaborators were tested by previous study.

Eukaryotic cell lines

Policy information about cell lines

Cell line source(s)

CENP-A-deficient DT40 cells [CENP-A (-/Flox), Mer-Cre-Mer] were established in the previous study [Dev. Cell 42, 181-189 (2017)].
CENP-A-deficient DT40 cells expressing GFP-CENP-A or GFP-CENP-A(QD) were established in this study.
Drosophila S2 cells were purchased from Gibco.

Authentication

CENP-A-deficient DT40 cells expressing GFP-CENP-A or GFP-CENP-A(QD) were analyzed by southern blotting and western blotting to confirm deletion of the endogenous CENP-A gene.

Mycoplasma contamination

All cell lines were not tested for mycoplasma contamination.

Commonly misidentified lines
(See ICLAC register)

CENP-A-deficient DT40 cells expressing GFP-CENP-A or GFP-CENP-A(QD) are not listed in the ICLAC register (v9.0).

ChIP-seq

Data deposition

☒ Confirm that both raw and final processed data have been deposited in a public database such as GEO.

☒ Confirm that you have deposited or provided access to graph files (e.g. BED files) for the called peaks.

Data access links
May remain private before publication.

SAMD00132316 (S2_input) http://trace.ddbj.nig.ac.jp/BSSearch/biosample?acc=SAMD00132316
SAMD00132321 (gfpCA-QD2-5_GFP_SpI) http://trace.ddbj.nig.ac.jp/BSSearch/biosample?acc=SAMD00132321
SAMD00132324 (gfpCA-QD2-5_H4K20me1_SpI) http://trace.ddbj.nig.ac.jp/BSSearch/biosample?acc=SAMD00132324
SAMD00132318 (gfpCA-QD2-5_IgG_SpI) http://trace.ddbj.nig.ac.jp/BSSearch/biosample?acc=SAMD00132318
SAMD00132322 (gfpCA-QD3-1_GFP_SpI) http://trace.ddbj.nig.ac.jp/BSSearch/biosample?acc=SAMD00132322
SAMD00132325 (gfpCA-QD3-1_H4K20me1_SpI) http://trace.ddbj.nig.ac.jp/BSSearch/biosample?acc=SAMD00132325
SAMD00132319 (gfpCA-QD3-1_IgG_SpI) http://trace.ddbj.nig.ac.jp/BSSearch/biosample?acc=SAMD00132319
SAMD00132320 (gfpCA1-1_GFP_SpI) http://trace.ddbj.nig.ac.jp/BSSearch/biosample?acc=SAMD00132320
SAMD00132323 (gfpCA1-1_H4K20me1_SpI) http://trace.ddbj.nig.ac.jp/BSSearch/biosample?acc=SAMD00132323
SAMD00132317 (gfpCA1-1_IgG_SpI) http://trace.ddbj.nig.ac.jp/BSSearch/biosample?acc=SAMD00132317
SAMD00132315 (gfpCA1-1_input) http://trace.ddbj.nig.ac.jp/BSSearch/biosample?acc=SAMD00132315

Files in database submission

SAMD00132316 (S2_input) http://trace.ddbj.nig.ac.jp/BSSearch/biosample?acc=SAMD00132316
SAMD00132321 (gfpCA-QD2-5_GFP_SpI) http://trace.ddbj.nig.ac.jp/BSSearch/biosample?acc=SAMD00132321
SAMD00132324 (gfpCA-QD2-5_H4K20me1_SpI) http://trace.ddbj.nig.ac.jp/BSSearch/biosample?acc=SAMD00132324
SAMD00132318 (gfpCA-QD2-5_IgG_SpI) http://trace.ddbj.nig.ac.jp/BSSearch/biosample?acc=SAMD00132318
SAMD00132322 (gfpCA-QD3-1_GFP_SpI) http://trace.ddbj.nig.ac.jp/BSSearch/biosample?acc=SAMD00132322
SAMD00132325 (gfpCA-QD3-1_H4K20me1_SpI) http://trace.ddbj.nig.ac.jp/BSSearch/biosample?acc=SAMD00132325
SAMD00132319 (gfpCA-QD3-1_IgG_SpI) http://trace.ddbj.nig.ac.jp/BSSearch/biosample?acc=SAMD00132319
SAMD00132320 (gfpCA1-1_GFP_SpI) http://trace.ddbj.nig.ac.jp/BSSearch/biosample?acc=SAMD00132320
SAMD00132323 (gfpCA1-1_H4K20me1_SpI) http://trace.ddbj.nig.ac.jp/BSSearch/biosample?acc=SAMD00132323
SAMD00132317 (gfpCA1-1_IgG_SpI) http://trace.ddbj.nig.ac.jp/BSSearch/biosample?acc=SAMD00132317
SAMD00132315 (gfpCA1-1_input) http://trace.ddbj.nig.ac.jp/BSSearch/biosample?acc=SAMD00132315

Genome browser session
(e.g. UCSC)

Final submission

Methodology

Replicates

Two independent cell lines were used for ChIP-seq.

Sequencing depth

Single end read sequencing (100bp). We aimed at sequencing over 20 million reads per sample for ChIP-seq. The total number of reads and number of mapped reads are listed in "Data quality" column in this document.

Antibodies	mouse anti-H4K20 monomethylation (#CMA421)<br>rabbit anti-GFP (MBL) : (https://www.mblintl.com/products/598)
Peak calling parameters	Peak calling was not performed in all ChIP-seq samples.

Data quality

The number of reads and quality measures are listed below.

gfpCA1-1_GFP_SpI
MAPQ >= 30 : 12177917 (43.1%)
MAPQ < 30 : 297794 (1.1%)
MAPQ < 20 : 50749 (0.2%)
MAPQ < 10 : 4621 (0%)
MAPQ < 3 : 278 (0%)
Unmapped : 15714068 (55.6%)
Total : 28245428 (100%)

gfpCA-QD2-5_GFP_SpI
MAPQ >= 30 : 15298077 (59%)
MAPQ < 30 : 273959 (1.1%)
MAPQ < 20 : 48700 (0.2%)
MAPQ < 10 : 5918 (0%)
MAPQ < 3 : 346 (0%)
Unmapped : 10301573 (39.7%)
Total : 25928572 (100%)

gfpCA-QD3-1_GFP_SpI
MAPQ >= 30 : 18833028 (64.7%)
MAPQ < 30 : 304441 (1%)
MAPQ < 20 : 56736 (0.2%)
MAPQ < 10 : 7057 (0%)
MAPQ < 3 : 437 (0%)
Unmapped : 9919403 (34.1%)
Total : 29121104 (100%)

gfpCA1-1_H4K20me1_SpI
MAPQ >= 30 : 24784610 (84.2%)
MAPQ < 30 : 269052 (0.9%)
MAPQ < 20 : 51136 (0.2%)
MAPQ < 10 : 8182 (0%)
MAPQ < 3 : 611 (0%)
Unmapped : 4305532 (14.6%)
Total : 29419124 (100%)

gfpCA-QD2-5_H4K20me1_SpI
MAPQ >= 30 : 24784694 (85.2%)
MAPQ < 30 : 263943 (0.9%)
MAPQ < 20 : 46783 (0.2%)
MAPQ < 10 : 7538 (0%)
MAPQ < 3 : 626 (0%)
Unmapped : 3997834 (13.7%)
Total : 29101418 (100%)

gfpCA-QD3-1_H4K20me1_SpI
MAPQ >= 30 : 21079448 (84.6%)
MAPQ < 30 : 227058 (0.9%)
MAPQ < 20 : 44119 (0.2%)
MAPQ < 10 : 7020 (0%)
MAPQ < 3 : 519 (0%)
Unmapped : 3556420 (14.3%)
Total : 24914584 (100%)

Software

The sequence data were mapped to the Chicken Genome database (NCBI, Gallus_gallus-4.0), as well as to the Drosophila genome database (NCBI, Drosophila-dm6), with the Burrows-Wheeler Aligner version 0.6.2 mapping tool.
